# 5-Lipoxygenase Deficiency Impairs Innate and Adaptive Immune Responses during Fungal Infection

**DOI:** 10.1371/journal.pone.0031701

**Published:** 2012-03-20

**Authors:** Adriana Secatto, Lilian Cataldi Rodrigues, Carlos Henrique Serezani, Simone Gusmão Ramos, Marcelo Dias-Baruffi, Lúcia Helena Faccioli, Alexandra I. Medeiros

**Affiliations:** 1 Departamento de Análises Clínicas, Toxicológicas e Bromatológicas, Faculdade de Ciências Farmacêuticas de Ribeirão Preto, Universidade de São Paulo, Ribeirão Preto, São Paulo, Brazil; 2 Division of Pulmonary and Critical Care Medicine, Department of Internal Medicine, University of Michigan Health System, Ann Arbor, Michigan, United States of America; 3 Departamento de Patologia, Faculdade de Medicina de Ribeirão Preto, Universidade de São Paulo, Ribeirão Preto, São Paulo, Brazil; 4 Departamento de Ciências Biológicas, Faculdade de Ciências Farmacêuticas, Universidade Estadual Paulista “Júlio de Mesquita Filho”, Araraquara, São Paulo, Brazil; University of Birmingham, United Kingdom

## Abstract

5-lipoxygenase-derived products have been implicated in both the inhibition and promotion of chronic infection. Here, we sought to investigate the roles of endogenous 5-lipoxygenase products and exogenous leukotrienes during *Histoplasma capsulatum* infection *in vivo* and *in vitro*. 5-LO deficiency led to increased lung CFU, decreased nitric oxide production and a deficient primary immune response during active fungal infection. Moreover, *H. capsulatum*-infected 5-LO^−/−^ mice showed an intense influx of neutrophils and an impaired ability to generate and recruit effector T cells to the lung. The fungal susceptibility of 5-LO^−/−^ mice correlated with a lower rate of macrophage ingestion of IgG-*H. capsulatum* relative to WT macrophages. Conversely, exogenous LTB4 and LTC4 restored macrophage phagocytosis in 5-LO deficient mice. Our results demonstrate that leukotrienes are required to control chronic fungal infection by amplifying both the innate and adaptive immune response during histoplasmosis.

## Introduction

*Histoplasma capsulatum* is a dimorphic, facultative, intracellular fungal pathogen ingested by resident cells such as alveolar macrophages and dendritic cells and by neutrophils when these inflammatory cells are recruited to the site of infection. The immune response against *H. capsulatum* is mediated by phagocytes, neutrophils, and CD4^+^ and CD8^+^ T cells [1]. The clearance of the fungus is associated with Th1-related cytokines, including IL-12, IFN-γ, TNF-α, and GM-CSF, which are essential for the development of a protective immune response in *H. capsulatum*-infected mice [2], [3], [4]. Fungal clearance is also associated with an overproduction of lipid mediators, such as leukotrienes (LTs), by phagocytes [5]. LTs are bioactive lipids derived from the 5-lipoxygenase (5-LO) pathway of arachidonic acid (AA) metabolism. The 5-LO-activating protein (FLAP) activates 5-LO that then oxygenates AA to form LTA_4_ [6]. This intermediate can be hydrolized to form LTB_4_ by LTA_4_ hydrolase or LTC_4_ synthase, which catalyzes the conjugation with glutathione to form the LTC_4_ that will be formed into LTD_4_ and LTE_4_, collectively known as cysteinyl LTs (CysLTs) [7]. 5-LO metabolites are known for their ability to function as neutrophil chemoattractants (LTB_4_) and for their effects on smooth muscle contraction during asthma (CysLTs). Currently, the relative roles of LTs in amplifying the innate and adaptive immune responses are not well understood. While it's been shown that endogenous and exogenous LTs enhance macrophage antimicrobial effector function and secretion of pro-inflammatory molecules [5], [8], [9], [10], other 5-LO derived products, such as lipoxins, may limit *Mycobacterium tuberculosis* [11] and *Trypanosoma cruzi* [12] infection. Thus, the specific role of 5-LO products in modulating chronic fungal infection remains to be fully understood. Among their many biological functions, LTs stimulate leukocyte migration and activation [13], microbial phagocytosis and killing [14], [15] and the chemotactic activity for *in vitro*-activated effector CD4^+^ and CD8^+^ T cells [16], [17] and γδ T lymphocytes [18]. Moreover, numerous *in vitro* and *in vivo* models have revealed a protective role for endogenous LTs, particularly during bacterial peritonitis and tuberculosis, parasitic and HIV infection [19]. We have recently demonstrated an important role for LTs in the primary and secondary immune responses against *H. capsulatum* [5], [20]. 5-LO deficiency impairs both the recruitment and activation of memory T cells following immunization against *H. capsulatum* [20]. Moreover, we have previously shown that pharmacological inhibition of LTs hinders host defense mechanisms during *H. capsulatum* infection. However, due to the off-target effects of LT inhibitors, which are associated with the partial inhibition of LTs, the precise role of 5-LO metabolites still remains to be determined. Here, by employing a genetic approach, 5-LO deficient mice (5-LO^−/−^) were used to demonstrate the role of endogenous LTs in histoplasmosis *in vivo* and *in vitro*. We demonstrate that LTs play an important role in host defense against *H. capsulatum* through the modulation of nitric oxide (NO) production, phagocytosis and effector cell recruitment.

## Results and Discussion

### 5-LO deficiency impairs *H. capsulatum* clearance and animal survival

**Figure 1:**
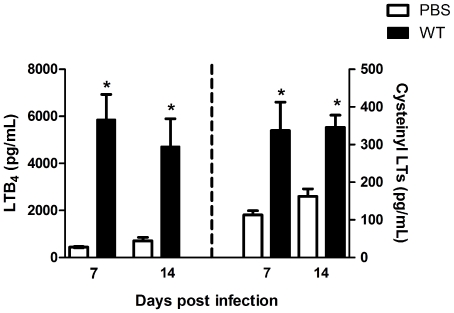
LTB_4_ and CysLTs production in lung tissue. Enzyme immunoassay quantification of LTB_4_ and CysLTs concentrations in lungs from mice that had received either an i.t. PBS injection (uninfected) or an i.t. infection with *H. capsulatum*. Data are presented as the mean ± SEM and are representative of one of two independent experiments (n = 6). *, p<0.05 vs. PBS.

**Figure 2:**
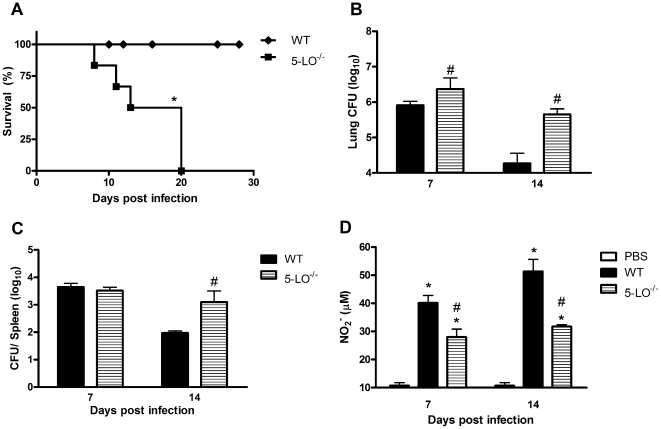
Effect of 5-LO deficiency on survival, fungal burden and NO_2_^−^ production. (A) 5-LO^−/−^ and WT mice were infected i.t. with 3×10^6^ yeast *H. capsulatum* and survival was monitored for 30 days (n = 6). CFU numbers in lungs (B) and spleen (C) were evaluated at 7 and 14 days post *H. capsulatum* infection. (D) NO_2_^−^ levels were quantified in the supernatant of lung homogenates at different time points using a Griess reaction. Data are expressed as the mean ± SEM from one experiment representative of a total of two experiments (n = 6). *, p<0.05 vs. PBS; #, p<0.05 vs. WT.

To determine if LTs are indeed required for host defense during *H. capsulatum* infection, we initially sought to investigate whether LTs are produced in the lungs of WT sv129-infected animals. Our results show that *H. capsulatum* infection induced LTB_4_ and CysLTs production at 7 and 14 days after infection when compared to uninfected mice (Figure 1). Moreover, we observed that greater amounts of LTB_4_ are produced relative to CysLTs during fungal infection. We then speculated whether the genetic deletion of the LT-generating enzyme would affect both survival and fungal load in murine models. Figure 2A shows that during the 30 days of the infection, 5-LO^−/−^ mice exhibited 100% mortality by day 20 whereas 100% of WT mice survived the infection. Next, we attempted to investigate if the increased mortality was due to higher fungal numbers in the 5-LO deficient lung. We observed that the fungal burden of 5-LO^−/−^ mice was significantly higher than WT mice at day 7 and was more pronounced at 14 days post-infection at the site of local infection (lung) and in the spleen (Figure 2B and 2C). Our data show that 5-LO metabolites are key mediators involved in the control of *H. capsulatum* infection by inhibiting fungal dissemination to other organs.

### LT-enhanced fungicidal activity in the lung is associated with nitric oxide (NO) generation

NO is a key microbicidal molecule involved in the control of *H. capsulatum* infection [21]. In addition, previous studies have shown that LTs enhance NO production in macrophages infected with protozoan parasites or macrophages stimulated with TLR agonists [22], [23], [24]. We next assessed whether increased susceptibility of 5-LO^−/−^ mice could be associated with lung NO production during fungal infection. Indeed, while *H. capsulatum* infection increased NO production in the lungs of WT mice at 7 days post-infection, 5-LO deficiency decreased NO production by ∼30% at day 7 and ∼50% at day 14 in the lungs of infected 5-LO^−/−^ mice as compared to infected WT mice (Figure 2D).

The low levels of NO in 5-LO−/− mice may be related to a deficiency in the production of 5-LO metabolites, such as LTs and lipoxins. The inhibition of NO synthesis by LT inhibitors or receptor antagonists has been demonstrated previously in other experimental models, such as *T. cruzi* [24], *M. tuberculosis* [8], and VSV encephalitis infection [10]. The predominance of neutrophils in the lungs of 5-LO^−/−^ infected mice suggests that 5-LO products may interfere either directly or indirectly with the synthesis of NO by neutrophils. In addition, the low levels of NO in the lungs of infected 5-LO^−/−^ mice could be explained by the predominance of neutrophils over macrophages. Macrophages are able to produce higher amounts of NO in the presence of inflammatory stimuli than neutrophils [25]. Therefore, our data suggest that low levels of NO, either by the predominance of neutrophils or through the modulation of NO metabolites by 5-LO products, could help explain the increased susceptibility to infection of 5-LO^−/−^ animals.

### LT-deficiency exacerbates the inflammatory response in the lung

**Figure 3:**
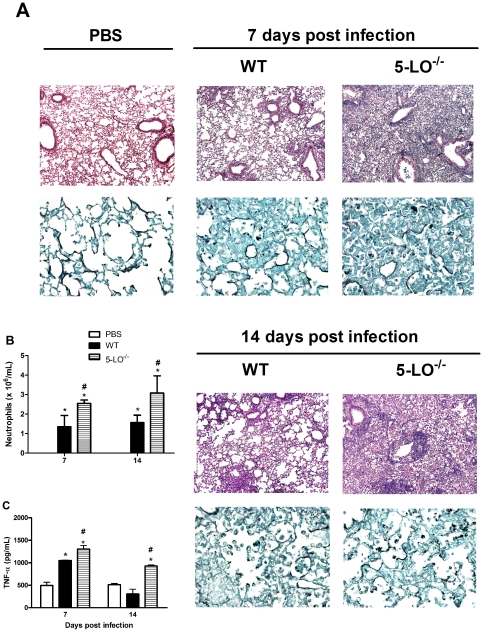
5-LO deficiency increases the inflammatory response in the lung. Representative lung sections from WT and 5-LO^−/−^ mice infected with *H. capsulatum* (A). Hematoxylin-eosin staining for leukocytes (magnifications ×100) and GMS staining for yeast cells (black arrow) (magnifications ×400). (B) Neutrophils recruitment from lung parenchyma (C) TNF-α production from homogenized lungs. Cells and cytokines were obtained as described in the *Material and Methods* section from mice after i.t. injection of PBS or i.t. infection with *H. capsulatum*. Cells were enumerated and identified after Rosenfeld staining, and TNF-α levels were determined by ELISA. Data are expressed as the mean ± SEM from one experiment representative of a total of two experiments (n = 6). *, p<0.05 vs. PBS; #, p<0.05 vs. WT.

To understand the increased fungal susceptibility of 5-LO^−/−^ mice, we examined the lungs using histopathological analysis. We observed an intense amount of inflammatory infiltrates in the lungs from WT infected-mice at days 7 and 14 post-infection, with a higher recruitment of neutrophils and mononuclear cells than the PBS-treated group (Figure 3A and B). Alternatively, lung tissue from 5-LO^−/−^ mice presented with an intense leukocyte infiltration with a predominance of neutrophils at days 7 and 14 post-infection as compared with WT mice (Figure 3A and B). Moreover, the strong influx of neutrophils in 5-LO^−/−^ mice was associated with high levels of TNF-α (Figure 3C). The intense neutrophil recruitment observed in 5-LO^−/−^ mice infected with *H. capsulatum* corroborates our previous findings [5]. The higher levels of TNF-α observed in 5-LO deficient mice and the exacerbation of neutrophil recruitment could be explained by the opposing effects of leukotrienes and lipoxins. While LTB_4_ enhances neutrophil recruitment and protects neutrophils from apoptosis, lipoxins enhance neutrophil efferocytosis by macrophages [26], [27]. Thus, the higher neutrophil recruitment to the lungs of 5-LO^−/−^ mice may be due to a lack of apoptotic cell clearance by macrophages due to the presence of lipoxin and an increase in apoptotic cells in the absence of LTB_4_. Moreover, the exacerbated neutrophil recruitment to the lung of 5-LO deficient infected mice may be associated with decreased levels of NO due to the modulation of chemotactic mediators other than LTs. Peritoneal macrophages from animals deficient in iNOS produce increased amounts of MCP-1 by promoting increased expression of CC chemokine receptors, which favors the efficient recruitment of neutrophils [28].

Even though neutrophils are important for the control of *H. capsulatum* infection, this cell type is known to exhibit more intense fungistatic versus fungicidal activity [29], [30], [31]. Based on this observation, we suggest that the inability of neutrophils to perform fungicidal or fungistatic functions may be impaired due to the absence of several factors, including LT synthesis. Mancuso et al. [32] showed that neutrophil phagocytosis of *Klebsiella pneumonia* is augmented by LTB_4_ and this mediator also participates in antimicrobial host defense [33]. However, other groups have shown that *M. tuberculosis*-infected 5-LO^−/−^ mice exhibit decreased neutrophil recruitment to the site of infection, decreased NOS2 mRNA levels and no modulation of TNF-α production [11]. The explanation for such contradictory effects may be related to the levels of lipoxin and leukotrienes induced by *H. capsulatum* and *M. tuberculosis* infection. The predominance or reduction of lipoxin and leukotrienes during the course of infection and the cross-talk between these mediators may explain contradictory results in both models. Whether lipoxins are important for *H. capsulatum* infection remains to be determined.

### Endogenous 5-LO metabolites increase phagocytosis of *H. capsulatum*

**Figure 4:**
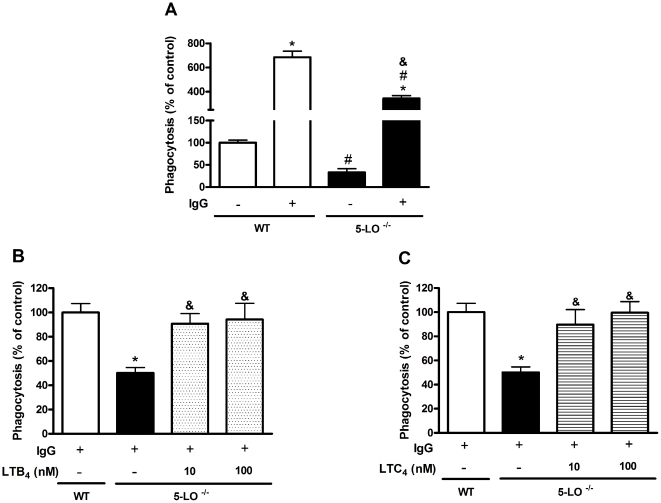
5-LO deficiency affects the ability of macrophages to phagocytose *H. capsulatum*. (A) PMs from WT and 5-LO^−/−^ mice were incubated for 1 h with a yeast:macrophage ratio of 1∶5 in the absence or presence of IgG. PMs were pretreated with LTB_4_ (B) and LTC_4_ (C) for 5 min before the addition of opsonized *H. capsulatum*. Phagocytosis was calculated as described in the *Material and Methods* section and was expressed as a percentage of the control. Data are expressed as the mean ± SEM from one experiment representative of a total of three experiments (n = 6). *, p<0.05 vs. control; #, p<0.05 vs. WT cells; & p<0.05 vs. 5-LO^−/−^ cells.

The effect of LTs on the enhancement of fungal host defense could be reflected by a diminished phagocytic capability of LT-deficient macrophages. It is known that LTs enhance phagocytosis of both IgG-opsonized bacteria and nonopsonized targets [34], [35]. Here, we determined the ability of 5-LO^−/−^ and WT peritoneal macrophages (PMs) to phagocytose *H. capsulatum*. WT macrophages exhibited higher rates of phagocytosis in IgG-opsonized *H. capsulatum* than for non-opsonized yeast. 5-LO deficient cells exhibited less phagocytosis of both opsonized and non-opsonized yeast when compared to WT macrophages (Figure 4A). These results demonstrated that endogenously produced 5-LO products are required for macrophage phagocytosis of *H. capsulatum* yeast. Bailie et al. [9] demonstrated that the increased susceptibility of 5-LO deficient mice during *K. pneumoniae* infection is associated with a deficiency in the phagocytic and bactericidal capacity of alveolar macrophages. Similarly, Morato-marques [35] showed that both LTB_4_ and CysLTs enhance both phagocytosis and killing of *C. albicans* by alveolar macrophages. In addition, exogenous LTs were able to restore the phagocytic ability of 5-LO^−/−^ alveolar macrophages infected with *K. pneumoniae* [14], [15]. Because our current results did not distinguish among the 5-LO products, we performed “add-back” experiments in which we added LTB_4_ or LTC_4_ to 5-LO^−/−^ macrophages and then measured phagocytosis. Pretreatment with LTB_4_ and LTC_4_ restored IgG-opsonized *H. capsulatum* phagocytosis by PMs from 5-LO^−/−^ mice in a dose-dependent manner (Figure 4B and 4C). We did not investigate whether LTs enhance *H. capsulatum* killing; however, we have previously observed that both LTB_4_ and CysLTs enhance the response to different pathogens. We demonstrated that LTB_4_ enhances the defense against *K. pneumoniae* and *C. albicans* in a manner dependent on ROI secretion; depletion of *L. amazonensis* is dependent on RNI generation by LTs. Because an effective immune response against *H. capsulatum* is mainly dependent on RNI production [36], we speculated that LTB_4_ could enhance NO secretion, which would further deplete *H. capsulatum*. The decreased NO synthesis observed in the Figure 2D reinforces our hypothesis. Resident macrophages are strategically distributed throughout various organs to maintain immunosurveillance through the phagocytosis, killing, and secretion of regulatory molecules, such as cytokines and lipid mediators. Since *H. capsulatum* is a facultative intracellular pathogen, our data may suggest that the inhibition of phagocytic ability may favor the proliferation of *H. capsulatum* outside of macrophages and could explain the increased CFU we observed in the lungs and spleens of 5-LO^−/−^ mice.

Our results show that among all 5-LO products produced during fungal infection, LTB_4_ and LTC_4_ improve the phagocytosis of opsonized *H. capsulatum* by peritoneal macrophages.

### Effect of 5-LO deficiency in recruitment of effector T cells

**Figure 5:**
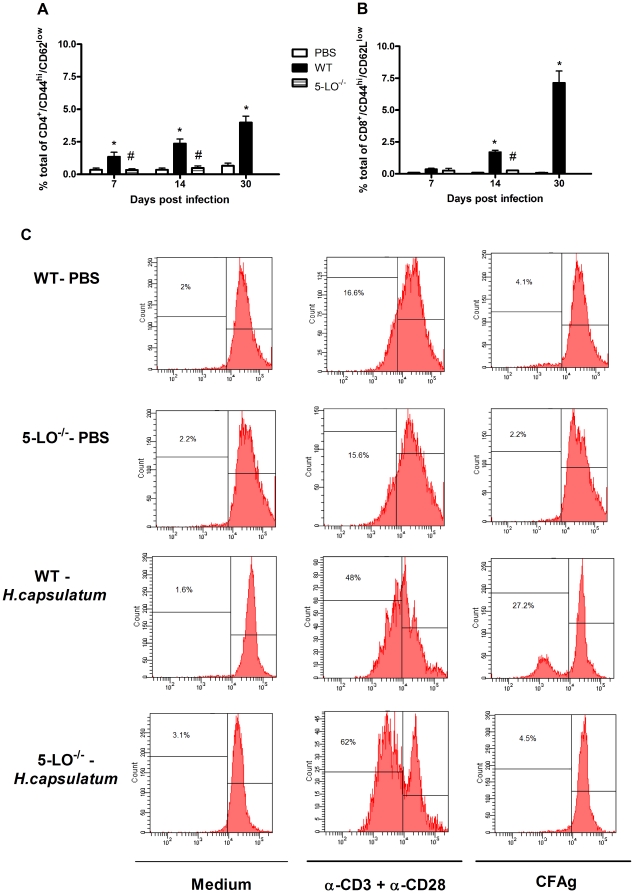
Deficiency of 5-LO impairs T cell recruitment to the lung. Cells were obtained as described in the *Material and Methods* section from mice after i.t. injection of PBS or i.t. infection with *H. capsulatum*. The lymphocyte population was gated for forward/side scatters and analyzed the percentage of T cells expressing a phenotype effector (CD44^high^/CD62^low^). (A) CD4^+^ T cells, CD8^+^ T cells (B) and T cell proliferation(C). Data are presented as the mean ± the SEM from three experiments. *, WT and 5-LO^−/−^ vs. PBS; #, WT vs. 5-LO^−/−^. p<0.05.

Previous studies have shown that LTs are important chemotactic factors for CD4^+^ [16] and CD8^+^ [17] T cells and that this recruitment depends on the expression of the BLT_1_ receptor. Next, we assessed whether *H. capsulatum*-infected 5-LO^−/−^ mice demonstrated defective leukocyte recruitment to the site of infection. Other than the importance of innate effector mechanisms that control fungal infection, a protective immune response against *H. capsulatum* also depends on the activation of antigen-specific CD4^+^ and CD8^+^ T cells [1]. We next explored the role of endogenous LTs in the recruitment of CD4^+^ and CD8^+^ effector T cells during *H. capsulatum* infection. The recruited population of effector CD4^+^ and CD8^+^ T cells was CD44^high^ and CD62^low^, and the number of CD4^+^ and CD8^+^ T cells increased progressively during infection in WT animals when compared with the PBS-treated group. In contrast, 5-LO deficiency blunted T cell recruitment to the lungs of infected 5-LO^−/−^ mice (Figure 5A and 5B).

Islam et al. [37] also showed that LTB_4_-BLT1 is a chemoattractant for CD4^+^ and CD8^+^ effector and memory T cells. Furthermore, we have shown that recruitment or activation of memory T cells (CD4^+^ and CD8^+^) during the secondary immune response against *H. capsulatum* was suppressed and was associated with an increased susceptibility for 5-LO deficient mice [20]. To determine whether the absence of effector T cells was due to decreased recruitment or generation of these cells, we performed proliferation assays using CFSE dilution of spleen cells from uninfected WT and 5-LO^−/−^ mice (PBS) and *H. capsulatum*-infected mice. As shown in Figure 5C, spleen cells from WT and 5-LO^−/−^ PBS and *H. capsulatum*-infected mice proliferate *in vitro* with α-CD3 and α-CD28 stimulation (polyclonal). Interestingly, only spleen cells from WT infected mice proliferated after stimulation with cell-free antigens (CFAgs, antigen-specific stimulation). Taken together, our results suggest that the increased mortality and CFU number in 5-LO^−/−^ mice may be associated with a deficiency in the activation and proliferation of antigen-specific T cells in the draining lymph node and the recruitment of these cells to the site of infection. The mechanisms underlying the role of LTs in T cell proliferation remain to be determined; however, some possibilities include the activation of Src and Syk kinases, which are known to be important for T cell proliferation and are also activated by LTB_4_ [38].

In summary, we demonstrate that inhibition of the 5-LO enzyme resulted in enhanced susceptibility to pulmonary fungal infection, which is associated with lung fungal persistence, decreased NO production, decreased phagocytic capacity and impaired proliferation and/or activation of effector T cells. These findings indicate that LTs are essential mediators that enhance the innate and adaptive immune response in the context of chronic infections such as histoplasmosis.

## Materials and Methods

### Mice

5-LO deficient or knockout (5 LO^−/−^) (129-Alox5^tmlfun^) mice and strain-matched wild-type (WT) sv129 mice (6–8 weeks old) were obtained from The Jackson Laboratory (Bar Harbor, ME, USA) and were bred in the Faculdade de Ciências Farmacêuticas de Ribeirão Preto (Universidade de São Paulo, Brazil). All experiments were approved and conducted in accordance with the guidelines of the Animal Care Committee of the University. Infected animals were kept in biohazard facilities and were housed in cages within a laminar flow safety enclosure under standard conditions.

### Preparation of *H. capsulatum* and infection of mice

The *H. capsulatum* strain used in this study was isolated from a human pulmonary clinical isolate at the Hospital das Clínicas from Faculdade de Medicina de Ribeirão Preto (Universidade de São Paulo, Brazil). Live mycelia were obtained by fungal culture at 25°C on Sabouraud dextrose agar (Difco, Detroit, MI, USA) for 2 months, and conidia morphology was observed. Later, mycelia were grown in BHI blood agar at 37°C for 7–15 days to convert them to yeast form and morphology was also confirmed.

Yeast cells were used when their viability was ≥90% based on fluorescein diacetate and ethidium bromide. The infection was performed as described previously [20]. Briefly, mice were anesthetized with tribromoethanol 2.5%, restrained on a small board and infected by the intratracheal route. Animals received either 100 µl phosphate buffered saline (PBS) or 3×10^6^ viable *H. capsulatum* yeast in 100 µl PBS (sublethal inoculum of *H. capsulatum*).

### Quantitation of fungal load in the spleen and lungs

Recovery of *H. capsulatum* was performed as previously described by Sá-Nunes et al. [39]. Three serial dilution was made and 0.2 ml of the dilution was plated on a BHI-agar-blood slant. The fungal burden was counted after incubation at 37°C for 21 days. The results are expressed as colony forming units (CFU) per lung and spleen.

### Quantitation of NO

NO production was determined by measuring the amount of nitrite (NO_2_^−^) in lung homogenates, obtained as described above, using the Greiss reaction as previously described [5].

### Measurement of LTs and cytokine

Lungs were removed on days 7 and 14 post-infection to measure LTB_4_, CysLTs and TNF-α. Briefly, tissue was homogenized (Mixer Homogenizer, Labortechnik, Germany) in 2 ml of RPMI1640, centrifuged and stored at −70°C until assayed. A specific enzyme immunoassay was used to quantify LTB_4_ and CysLTs (LTC_4_/D_4_/E_4_, Cayman Chemical, Ann Arbor, Mich.) according to the manufacturer's instructions [20]. Commercially available ELISAs were used to measure TNF-α (R&D Systems, Minneapolis, MN). The sensitivity of the assay was <10 pg/ml.

### Histopathologic analysis

Lungs were removed on days 7 and 14 post-infection, and tissues were fixed in 10% formalin, embedded in paraffin, cut into four to five µm sections and stained with haematoxylin and eosin (HE) and Grocott's methanemine silver (GMS). Analysis of these sections was performed with a video camera (Leica Microsystems Ltd., Heebrugg, Switzerland) applied to a Leica microscope DMR (Leica, Microsystems GmbH, Wetzlar, Germany) that was attached to a computer. Images were processed by Leica QWin software (Leica Microsystems Image Solutions, Cambridge, UK).

### Peritoneal macrophages (PMs) isolation and culture

PMs were obtained from control 5-LO^−/−^ and WT mice by washing peritoneal cavities with 5 ml of ice-cold phosphate buffered saline (PBS). The cells were centrifuged (160×*g*, 10 min, 4°C), resuspended in RPMI 1640 medium, and were adjusted to 2×10^6^ cells/ml. The percentage of macrophages was determined microscopically using a modified Rosenfeld stain, where a typical experiment yielded ∼95% macrophages. Cells were cultured overnight in RPMI containing 10% fetal bovine serum and were washed twice the next day with warm free serum medium so that non-adherent cells could be removed.

### Fungal phagocytosis assays

Phagocytosis assays were assessed using an adapted protocol that was previously described [40]. Briefly, *H. capsulatum* yeast was opsonized with 10% heat-inactivated specific immune serum (IgG) for 40 min at 37°C. The serum containing specific IgG without complement was prepared by heating to 65°C for 30 minutes in water bath. After opsonization, yeast cells were washed and labeled with FITC (AMRESCO) for 1 h at 37°C. IgG-opsonized FITC-labeled *H. capsulatum* was further diluted in RPMI incubated with PMs at a ratio of 1∶5 (yeast cell: macrophage). After a 1 h incubation in the dark (37°C, 5% CO2), uningested yeasts cell were washed with phosphate buffered saline (PBS), and residual extracellular FITC was quenched with trypan blue (250 mg/ml; Molecular Probes) for 1 min. Fluorescence was determined with a microplate fluorometer (485ex/535em,SPECTRAFluor Plus; Tecan, Research Triangle Park, NC). In some experiments, cells were pretreated for 5 min with LTB_4_ or LTC_4_ (Cayman Chemical, Ann Arbor, Mich.) diluted in RPMI before the addition of IgG-opsonized FITC labeled *H. capsulatum*. The results are expressed as a percentage of the control.

### Spleen cells isolation and proliferation assay

Spleens from infected mice (7 days post-infection) and age-matched uninfected control mice were aseptically removed and minced, and the released cells were washed three times in RPMI 1640 (Gibco BRL, Grand Island, USA). Cells were resuspended at 5×106 cells/ml in RPMI supplemented with 10% fetal bovine serum (Gibco BRL), penicillin (100 U/ml, Gibco BRL) and streptomycin (100 µg/ml, Gibco BRL) and were dispensed into 96-well flat- bottom microtiter plates in 0.1 ml. For polyclonal stimulation, α-CD3, α-CD28 (2 µg/ml, Sigma Chemical Co., St. Louis, USA) and specific stimulation, cell-free antigens from *H. capsulatum* (CFAgs) (50 µg/ml) [39] were added to wells (0.1 ml) in triplicate and cultured for 72 h at 37°C. Splenic cell proliferation was measured by dilution of intracellular CFSE staining as detected by flow cytometry (FACSort™ - BD Bioscience).

### Leukocytes isolated from lung parenchyma

Approximately 7 and 14 days following infection, the lung was removed and total cells were obtained by enzymatic digestion as described previously [41]. Leukocyte numbers and differential counts for neutrophils were obtained as described previously [42].

### Flow cytometric analysis

Lung leukocytes were adjusted to a concentration of 5×10^5^ cells/100 µL, and FcγRs were blocked by the addition of unlabeled anti-CD16/32. Leukocytes were stained with anti-CD4 mAb (PerCP- Cy 5.5), -CD8 mAb(PerCP Cy.5.5), -CD44 mAb (FITC) and -CD62 L (PE) mAb murine specific and isotype controls for 30 min at 4°C (BD Pharmingen) as previously described [20]. The results were calculated by determining the percentage of total CD4^+^ or CD8^+^ T cells with an effector phenotype (CD4^+^CD44 h^igh^CD62 L^low/neg^ or CD8^+^CD44 h^igh^CD62 L^low/neg^). T cell immunophenotyping was performed using FACSort (BD Biosciences) and CellQuest software, and T cell proliferation was analyzed using FACSCanto (BD Biosciences) and FACSDiva software.

### Statistical analysis

The data are presented as the mean ± SEM. Comparisons were performed using an ANOVA followed by the Bonferroni test by the Prism 4.0 statistical program (GraphPad Software, San Diego,CA). Differences in survival were analyzed by the log rank test. Values of p<0.05 were considered statistically significant.
